# Efficacy of intraarticular botulinum toxin A and intraarticular hyaluronate plus rehabilitation exercise in patients with unilateral ankle osteoarthritis: a randomized controlled trial

**DOI:** 10.1186/1757-1146-7-9

**Published:** 2014-02-06

**Authors:** Shu-Fen Sun, Chien-Wei Hsu, Huey-Shyan Lin, Yi-Jiun Chou, Jun-Yang Chen, Jue-Long Wang

**Affiliations:** 1 Department of Physical Medicine and Rehabilitation, Kaohsiung Veterans General Hospital, Kaohsiung, Taiwan; 2 National Yang-Ming University School of Medicine, Taipei, Taiwan; 3 Department of Internal Medicine, Kaohsiung Veterans General Hospital, Kaohsiung, Taiwan; 4 School of Nursing, Fooyin University, Kaohsiung, Taiwan; 5 Department of Health, Statistical Consultant of Research and Development, Kaohsiung City Government, Kaohsiung, Taiwan; 6 Department of Orthopedic Surgery, Kaohsiung Veterans General Hospital, Kaohsiung, Taiwan

**Keywords:** Ankle, Osteoarthritis, Botulinum toxin, Hyaluronate, Intraarticular injection

## Abstract

**Background:**

There was an increasing requirement for novel treatments of osteoarthritis (OA). The aim was to compare the efficacy of intraarticular Botulinum toxin type A (BoNT-A) and intraarticular hyaluronate plus rehabilitation exercise in patients with ankle OA.

**Methods:**

This was a prospective, randomized, assessor-blinded study with a 6-month follow-up period, conducted in the outpatient rehabilitation department at a university-affiliated tertiary care medical center. Seventy-five patients with symptomatic ankle OA (Kellgren-Lawrence grade 2) were randomized to receive either a single 100-unit BoNT-A injection into the target ankle (n = 38) or a single hyaluronate injection plus 12 sessions of rehabilitation exercise (30 minutes/day, 3 times/week for 4 weeks) (n = 37). The primary outcome measure was the Ankle Osteoarthritis Scale (AOS). Secondary outcome measures included American Orthopedic Foot and Ankle Society (AOFAS) Ankle/Hindfoot Score, visual analog scale (VAS) for ankle pain, single leg stance test (SLS), Timed “Up-and-Go” test (TUG), consumption of rescue analgesics and global patient satisfaction.

**Results:**

There were no significant between-group differences in total AOS scores, pain subscale and disability subscale scores (adjusted mean difference AMD = -0.2, 95% CI = (-0.5, 0.2), p = 0.39; AMD = -0.1, 95% CI = (-0.5, 0.3), p = 0.57; AMD = -0.2, 95% CI = (-0.6, 0.2), p = 0.36). The 2 groups showed no significant differences in AOFAS, VAS, SLS, TUG scores and consumption of rescue analgesics at each follow-up visit, except that the hyaluronate group improved more in SLS than the BoNT-A group at 1-month follow-up. Patients’ satisfaction rate was high, with no serious adverse events. There was no difference in adverse events between the two groups (*p* = 1.00).

**Conclusions:**

Treatment with intraarticular BoNT-A or hyaluronate injection plus rehabilitation exercise was associated with improvements in pain, physical function and balance in patients with ankle OA. These effects were rapid at 2 weeks and might last for at least 6 months. There was no difference in effectiveness between the two interventions.

**Trial registration:**

The trial was registered at clinical trials.gov (Registry number NCT01760577).

## Background

Ankle osteoarthritis (OA) can cause substantial pain and functional limitations. Symptomatic ankle OA is found in less than 1% of the adult population and recent research has indicated patients are being diagnosed with ankle OA with increasing frequency [1,2]. Current treatment options include analgesics, non-steroidal anti-inflammatory medication (NSAIDs), weight loss, physical therapy, exercise, activity modification, assistive devices, local injections, and surgery. Although many cases can be treated successfully with surgery, some patients are either not good candidates for surgery or prefer not to have surgery. Long-term effective and safe alternative treatments that may reduce pain and improve function yet avoid the toxic effects of medications should be fully exploited. The potential treatment options include intraarticular injection of Botulinum toxin type A (BoNT-A) and hyaluronate injection plus rehabilitation exercise.

BoNT-A has been used clinically for its muscle paralyzing effects, but there is increasing evidence to support using it in pain modulation [3,4]. Recent pilot studies report that BoNT-A injection into painful joints of patients with various types of arthritis leads to significant improvement in pain and function and is safe to use [5-8]. The initial effects for BoNT-A were encouraging because two thirds of the patients had more than 50% reduction in pain and was associated with significant functional improvement [8]. To date, there is no published literature that has prospectively evaluated the efficacy of intraarticular BoNT-A in the treatment of ankle OA.

Hyaluronate, a high-molecular-weight polysaccharide and a major component of the synovial fluid, acts as a lubricant and shock absorber and helps to maintain the structural and functional characteristics of the cartilage matrix. It also inhibits the formation and release of prostaglandins, induces proteoglycan aggregation and synthesis, and modulates the inflammatory response [9,10]. There is only limited published literature relating its use in ankle OA and dosing in the ankles remains an area for discussion [11-15]. Previous studies showed that 3 or 5 weekly injections of hyaluronate may improve pain and physical function in patients with ankle OA [11-13]. Theoretically, the ankle joint is much smaller than the knee, thus it is likely that less hyaluronate is needed to be effective in the ankle. Exercise has been proven to be an important factor in maintaining strength and flexibility and slowing the onset of debilitation in OA [16]. The advantage of exercise may be its noninvasive nature being preferred by both the patients and the physicians. No study to date has examined efficacy of intraarticular hyaluronate plus rehabilitation exercise in patient with ankle OA.

There is an increasing requirement for novel treatments of OA as the aging population is expanding with many patients who are unable to undergo joint surgery. Effective therapy has been a key therapeutic challenge. The purpose of this study was to conduct a randomized controlled trial to compare the efficacy of intraarticular BoNT-A and intraarticular hyaluronate plus rehabilitation exercise in patients with ankle OA.

## Methods

### Participants

Patients in this study were referred from our outpatient orthopedic department with a diagnosis of ankle OA. All patients fulfilled the following inclusion criteria: (1) an age of 20–85 years; (2) unilateral ankle pain that had lasted for at least 6 months, with no significant benefit from conservative treatment or with an inability to tolerate the side effects of medications; (3) ankle radiographs taken within 6 months equivalent to grade 2 on the Kellgren-Lawrence grading system [17]; (4) a current total Ankle Osteoarthritis Scale (AOS) score of (described below) of >3 and ≤ 9 (possible range, 0–10) [18]; (5) a normal activity level—i.e., not bedridden or confined to a wheelchair, and able to walk 30 meters without the aid of a walker, crutches or cane; and (6) no changes in shoes or orthotic devices during the study period.

Exclusion criteria included pregnancy or lactation in women; lower leg trauma other than ankle trauma; previous surgery involving the spine, hip or knee; presence of an active joint infections of foot or ankle; history of rheumatoid arthritis, gout, or any other inflammatory arthropathy, physical therapy involving the affected ankle within the previous 2 months; surgery involving the affected ankle within the previous twelve months; known allergy to chicken, egg or BoNT-A; steroid or hyaluronate injections in the ankle within the previous six months; treatment with anticoagulants or immunosuppressives; presence of other comorbidity (such as neoplasms, diabetes mellitus or recent trauma) or poor health status that would interfere with the assessments during the study.

The medical records for each patient were reviewed, and the etiology and duration of OA were recorded prior to the injection. The etiology was determined on the basis of the medical history, physical examination, and imaging studies. If no cause could be elucidated, the case was classified as primary OA.

### Study design

This was a prospective, assessor-blinded randomized controlled trial with a 6-month follow-up period, conducted in the outpatient rehabilitation department at a university-affiliated tertiary care medical center. The recruitment period was between May 2010 and November 2011. The study was approved by the institutional review board for human investigation (ethics approval number VGHKS99-CT4-19) and all participants provided signed informed consent before being enrolled in the study.

Intake of analgesics or NSAIDs was not permitted during the study period. Acetaminophen (500 mg), limited to 4 g/day was allowed as rescue medication. If the treatment dose was above the stipulated limit (acetaminophen 4 g/day), the patient was regarded as a clinical failure. Patients taking analgesics or NSAIDs stopped them at least 7 days before the preinjection assessment. Administration of acetaminophen was stopped at least 8 hours before the follow-up assessments. The administration of all analgesic medication during the study period was recorded on a diary card by the patient.

### Interventions

After completing the baseline survey within 1 week of entry into this study, the patients were randomized into 2 groups using block randomization in groups of four by a study assistant who did not participate in patients’ evaluation or treatment. The BoNT-A group received intraarticular injection of 100-unit of BoNT-A (Allergan, Inc, Irvine CA) reconstituted in 2 cc normal saline. The hyaluronate group received a single injection of 2 ml sodium hyaluronate (Hyalgan, molecular weight 500-730 kDa, Fidia Pharmaceutical Corporation, Italy), followed by 12 sessions of rehabilitation exercise for 30 minutes/day, 3 times per week for 4 weeks. The exercise program, commencing within 3 days of hyaluronate injection, included a series of ankle range of motion exercises, stretching exercises, isometric exercises and strengthening exercises for ankle muscles, and proprioceptive exercise (Additional file 1). Patients received instruction to continue the exercise program done in hospital at home for 30 minutes/day and a diary card for exercise was given to improve compliance at home. The home exercise program was taught by one physical therapist.

All the injections were performed by a single experienced physician, who took no part in the clinical assessment or in the data analysis. The patient was placed in the supine position, with the knee flexed and the feet flat on the plinth. Using aseptic procedures, the ankle joint was injected by inserting the needle 1 cm anterior to the distal medial malleolus and advancing the needle posteriorly and slightly superiorly toward the middle of the ankle joint above the talus. If an effusion was present, it was aspirated before injecting. In this study, the patients were not blinded to injection material.

### Outcome assessments

The clinical assessment was documented by a single assessor blinded to patient groups and intervention. These tests were conducted pre-injection and at 2 weeks, 1 month, 3 months, and 6 months post injection. Patients’ global satisfaction was assessed at 2 weeks, 1 month, 3 months, and 6 months post injection.

#### Primary outcome measures

The primary outcome measure was the total Ankle Osteoarthritis Scale (AOS) score, which is a validated patient-rated measure including a nine-item pain subscale and a nine-item disability subscale [18]. The range of possible scores for the AOS and its subscales is 0 to 10. A score of 0 represents no pain or disability and a score of 10 represents the worst pain or disability imaginable. The primary endpoint was 6 months.

#### Secondary outcome measures

Secondary outcome measures included the following:

(1) American Orthopedic Foot and Ankle Society (AOFAS) Ankle/Hindfoot Score was a 100-point scale that devoted 40 points to pain, 50 points to function and 10 points to alignment [19]. The maximum score of 100 points denoted no pain and normal function and alignment.

(2) The patient rated the intensity of average ankle pain in the previous week using a 10-cm horizontal visual analog scale (VAS) [20]. The VAS was marked in 1-cm increments from ‘no pain’to ‘worst pain’.

(3) Single-leg stance test (SLS) involved raising the unaffected foot, without touching it to the affected lower extremity, and maintaining balance for as long as possible. Each participant performed three trials, and the best result was recorded [21].

(4) A Timed “ Up-and-Go” test (TUG) measured functional mobility and the dynamic balance [22]. A patient was asked to rise from an armchair, walk 3 meters at a safe and comfortable pace, turn around, walk back to the chair, and sit down again. The total time (in seconds) required to complete this series of tasks was recorded.

(5) Patients rated their level of global satisfaction relative to the state before the treatment at each follow-up visit. This rating was based on a 0–6 7-point Likert scale ranging from completely dissatisfied (0), dissatisfied (1), somewhat dissatisfied (2), no change (3), somewhat satisfied (4), satisfied (5) to completely satisfied (6). In analysis, we took the global satisfaction as a dichotomous variable with 1 representing satisfied (point 4–6 in the 7-point scale) and 0 representing dissatisfied (point 0–3 in the 7-point scale).

(6) The administration of acetaminophen during the study period was recorded on a diary card by the patient.

(7) The safety of each injection was monitored by recording the occurrence of systemic and local adverse events on a diary card.

### Statistical analysis

Based on the Statistical Software Sample Power 2.0 and the statistical method used for the study hypothesis (there was a difference between the efficacy of two interventions), ANCOVA, the required sample size was 35 participants for each group (power = 0.8; alpha = 0.05; R^2^ of pretest to posttest 0.09; effect size 0.325).

All statistical procedures were conducted with the Statistical Package for the Social Sciences (version 12.0; SPSS Inc., Chicago, Illinois). Descriptive statistics such as mean, standard deviation, frequency and percentage were used to describe the participants’ demographic data in each group. Missing data were multiply imputed with regression methods and five iterations were used that intention-to-treat analysis can be conducted. The outcome variables used with multiple imputation included AOS, pain subscale, disability subscale, AOFAS Ankle/Hindfoot Score, VAS, SLS, TUG, acetaminophen consumption and patients’ global satisfaction, each at 2 weeks, 1 month, 3 months, and 6 months post injection. The predictor/independent variables used were in the following sequence: group, age, gender, body height, body weight, light or heavy worker, side of ankle injected, disease duration, and AOS, pain subscale, disability subscale, AOFAS Ankle/Hindfoot Score, VAS, SLS, TUG, acetaminophen consumption and patients’ global satisfaction, each at baseline, 2 weeks, 1 month, 3 months, and 6 months post injection. The analysis of covariance (ANCOVA) using baseline data as the covariate was used to detect the differences of the outcome variables (including AOS, AOFAS Ankle/Hindfoot Score, VAS, SLS, TUG, and analgesics consumption) between two groups at each post time point. Additionally, Fisher’s exact test was used to examine if there was significant difference in frequency of adverse events/satisfaction between the two groups. *p* values of less than 0.05 were regarded as significant.

## Results

**Figure 1:**
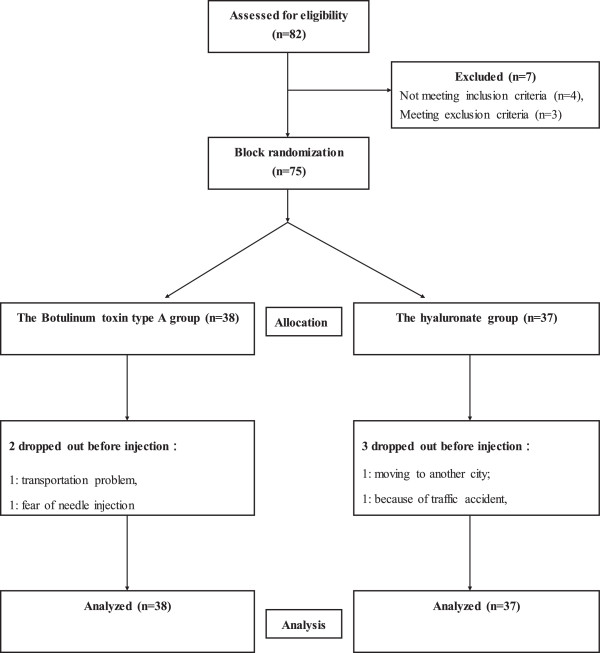
Flow diagram of patients through the trial.

Eighty-two patients were initially screened, seven subjects were excluded as four subjects did not meet inclusion criteria and three met the exclusion criteria. Seventy-five patients met all eligibility criteria and participated in the study (Figure 1). Five patients withdrew, two in the BoNT-A group because of transportation problems and fear of needle injection, three in the hyaluronate group because of moving to another city, traffic accident and poor compliance. Dropout of all these 5 patients occurred prior to injection treatment. From November 2010 to May 2012, a total of seventy patients completed the study (attrition rate 6.67%). Among the 75 patients who were originally in the study, ankle OA was attributed to primary OA in 21 patients. Secondary OA was noted in 54 patients. Table 1 summarizes the demographic data and patient characteristics in both groups.

**Table 1:** Demographic data and disease characteristics of the patients

**Characteristic**	**BoNT-A group (n = 38)**	**HA group (n = 37)**
Age (years)	49.5 ± 10.9	50.6 ± 10.3
Male, n (%)	23 (60.5)	23 (62.2)
Weight (kg)	67.5 ± 8.1	67.0 ± 10.5
Height (cm)	166.5 ± 6.5	165.1 ± 8.1
Etiology of OA, secondary OA (%)	27 (71.1)	27(73.0)
Light worker, n (%)	28 (73.7)	27 (73.0)
Side of ankle injected, left, n (%)	13 (34.2)	12 (32.4)
Disease duration (years)	5.6 ± 3.7	5.3 ± 4.0
Total AOS	4.9 ± 1.5	4.7 ± 1.0
Pain subscale	4.5 ± 1.3	4.5 ± 1.1
Disability subscale	5.2 ± 1.9	5.0 ± 1.3

Data are mean ± standard deviation or number (percentage).

*BoNT-A* = Botulinum toxin type A; *HA* = Hyaluronate; *OA* = Osteoarthritis; *AOS* = Ankle Osteoarthritis Scale.

### Intention-to-treat analysis

#### Primary outcome measures

The primary outcome measure was the total AOS. There were no significant differences between the 2 groups in total AOS scores, pain subscale and disability subscale scores (Table 2).

**Table 2:** Summary of primary outcome measures by data of multiple imputation

**Outcome**	**Baseline**		**2 weeks**		**1 month**		**3 months**		**6 months**	
	**BoNT-A**	**HA**	**BoNT-A**	**HA**	**BoNT-A**	**HA**	**BoNT-A**	**HA**	**BoNT-A**	**HA**
Total AOS^a^	4.9 ± 1.5	4.7 ± 1.0	2.7 ± 1.3	3.0 ± 1.3	2.5 ± 1.4	2.6 ± 1.2	2.5 ± 1.4	2.6 ± 1.2	2.6 ± 1.2	2.7 ± 1.0
A.M.D. (95% CI)			-0.3 (-0.8, 0.2)		-0.2 (-0.5, 0.2)		-0.2 (-0.6, 0.2)		-0.2 (-0.5, 0.2)	
*p*^b^			0.18		0.41		0.30		0.39	
Pain subscale^a^	4.5 ± 1.3	4.5 ± 1.1	2.5 ± 1.2	2.8 ± 1.3	2.3 ± 1.4	2.3 ± 1.0	2.3 ± 1.4	2.4 ± 1.1	2.4 ± 1.2	2.5 ± 1.1
A.M.D. (95% CI)			-0.3 (-0.8, 0.2)		-0.04 (-0.4, 0.3)		-0.2 (-0.6, 0.2)		-0.1 (-0.5, 0.3)	
*p*^b^			0.24		0.85		0.42		0.57	
Disability subscale^a^	5.2 ± 1.9	5.0 ± 1.3	3.0 ± 1.5	3.2 ± 1.4	2.8 ± 1.5	2.9 ± 1.7	2.8 ± 1.5	2.8 ± 1.4	2.9 ± 1.3	2.9 ± 1.1
A.M.D. (95% CI)			-0.3 (-0.8, 0.2)		-0.3 (-0.7, 0.2)		-0.2 (-0.7, 0.2)		-0.2 (-0.6, 0.2)	
*p*^b^			0.18		0.23		0.31		0.36	

NOTE: Values are the mean ± standard deviation; *BoNT-A* = Botulinum toxin type A; *HA* = Hyaluronate; *AOS* = Ankle Osteoarthritis Scale; *A.M.D.* = Adjusted mean difference; *CI* = Confidence interval.

^a^Higher scores represent worse pain or function.

^b^ANCOVA for comparing two groups in each posttest.

The primary endpoint was 6 months.

#### Secondary outcome measures

Table 3 provides a summary of secondary outcome measures at each follow-up visit. Results of AOFAS Ankle/Hindfoot Score, VAS, SLS, TUG tests and analgesics consumption showed no significant differences between the 2 groups, except that the hyaluronate group improved more in SLS than the BoNT-A group at 1-month follow-up.

**Table 3:** Summary of secondary outcome measures by data of multiple imputation

**Outcome**	**Baseline**		**2 weeks**		**1 month**		**3 months**		**6 months**	
	**BoNT-A**	**HA**	**BoNT-A**	**HA**	**BoNT-A**	**HA**	**BoNT-A**	**HA**	**BoNT-A**	**HA**
AOFAS Ankle/Hindfoot score	71.3 ± 11.6	70.0 ± 11.7	84.5 ± 8.8	82.9 ± 12.3	88.1 ± 12.1	88.1 ± 11.8	89.0 ± 7.3	88.1 ± 11.3	88.3 ± 7.2	86.4 ± 12.5
A.M.D. (95% CI)			1.4 (-2.5, 5.4)		-0.2 (-4.1, 3.8)		0.8 (-2.8, 4.4)		1.9 (-1.7, 5.5)	
*p*^b^			0.48		0.93		0.66		0.30	
VAS pain scale^a^	4.0 ± 1.8	3.9 ± 1.2	1.8 ± 1.2	1.8 ± 1.2	1.8 ± 1.3	1.7 ± 1.5	1.7 ± 1.2	1.7 ± 1.1	1.8 ± 0.9	1.7 ± 1.1
A.M.D. (95% CI)			-0.04 (-0.5, 0.5)		0.01 (-0.6, 0.6)		0.01 (-0.4, 0.5)		0.1 (-0.3, 0.5)	
*p*^b^			0.88		0.97		0.95		0.71	
SLS	25.0 ± 19.4	25.4 ± 16.0	34.1 ± 26.6	36.8 ± 22.5	36.9 ± 23.8	43.0 ± 18.6	36.8 ± 21.8	41.7 ± 19.6	36.5 ± 23.3	40.0 ± 20.0
A.M.D. (95% CI)			-2.3 (-6.3, 1.7)		-5.7 (-10.4, -1.0)		-4.6 (-9.7, 0.5)		-3.1 (-8.2, 2.1)	
*p*^b^			0.26		0.02*		0.08		0.24	
TUG^a^	8.4 ± 3.0	8.2 ± 2.5	7.2 ± 2.3	6.9 ± 2.4	6.5 ± 1.7	6.3 ± 1.9	6.6 ± 1.7	6.6 ± 2.0	6.8 ± 1.8	6.7 ± 2.1
A.M.D. (95% CI)			0.1 (-0.3, 0.5)		0.1 (-0.4, 0.5)		-0.1 (-0.6, 0.4)		-0.04 (-0.5, 0.5)	
*p*^b^			0.59		0.72		0.56		0.88	
Acetaminophen^a^ (tablets/week)	16.0 ± 5.4	17.0 ± 6.4	8.4 ± 5.2	8.9 ± 4.9	8.3 ± 4.3	8.5 ± 3.2	8.7 ± 4.9	9.3 ± 3.6	9.2 ± 4.9	9.5 ± 6.0
A.M.D. (95% CI)			-0.1 (-1.6, 1.5)		0.3 (-1.0, 1.5)		-0.1 (-1.5, 1.4)		0.3 (-1.1, 1.7)	
*p*^b^			0.93		0.67		0.94		0.66	

Values are the mean ± standard deviation; *AOFAS* = the American Orthopedic Foot and Ankle Society, *VAS* = Visual analog scale; *SLS* = single leg stance test; *TUG* = timed “Up-and-Go” test; The possible range for the AOFAS score was 0–100; *BoNT-A* = Botulinum toxin type A; *HA* = Hyaluronate; *A.M.D.* = Adjusted mean difference; *CI* = Confidence interval.

^a^Higher scores represent worse pain or function.; ^b^ANCOVA for comparing two groups in each posttest.

*p < 0.05.

Each of the two treatments resulted in a high rate of patient satisfaction (94.2% in the BoNT-A group and 94.1% in the hyaluronate group, at least somewhat satisfied) at the 6-month follow-up visit (Table 4).

**Table 4:** Satisfaction for the treatment of ankle osteoarthritis by data of multiple imputation

	**Satisfied**^**a**^		**Dissatisfied**^**b**^		**Comparison between groups**	
	**BoNT-A**	**HA**	**BoNT-A**	**HA**	***p***^**c**^	**OR (95% CI)**
2 weeks	34.4 (90.5)	33.4 (90.3)	3.6 (9.5)	3.6 (9.7)	1.00	1.03 (0.2, 4.7)
1 month	35.2 (92.6)	34.4 (93.0)	2.8 (7.4)	2.6 (7.0)	1.00	0.87 (0.1, 6.6)
3 months	38.0 (100.0)	37.0 (100.0)	0.0 (0.0)	0.0 (0.0)	-	-
6 months	35.8 (94.2)	34.8 (94.1)	2.2 (5.8)	2.2 (5.9)	1.00	1.08 (0.2, 7.4)

^a^Satisfied including somewhat satisfied, satisfied, and completely satisfied.

^b^Dissatisfied including completely dissatisfied, dissatisfied, somewhat dissatisfied, and no change.

^c^Fisher’s exact test.

Data are number (percentage).

BoNT-A = Botulinum toxin type A; HA = Hyaluronate; OR = Odds ratio; CI = Confidence interval.

#### Adverse effects

All patients tolerated the treatment well, with a low incidence of adverse events in both groups. Among the 70 patients who completed this study, transient injection-site reactions with mild to moderate pain or local swelling was reported by 2 (5.6%) patients in the BoNT-A group and 2 (5.9%) in the hyaluronate group. No severe or systemic adverse events were reported during the study period. There were no significant differences in adverse events between the two groups (*p* = 1.00).

## Discussion

This prospective study revealed that a single intraarticular injection of BoNT-A and a single hyaluronate injection plus rehabilitation exercise both resulted in improvements in pain, function and balance for patients with unilateral ankle OA. There were no significant differences between the 2 groups in most outcome variables. In both groups, the patients’ satisfaction rate was high with no serious adverse events. These effects were rapid at 2 weeks and could last for at least six months.

This study is the first prospective trial that evaluate efficacy of BoNT-A injection in patients with ankle OA. The results of using BoNT-A for pain relief in the ankle are consistent with those of previously studies using BoNT-A in the joints [5-8]. It is of interest that six months following the BoNT-A injections, the mean AOS pain subscale reduced from 4.5 ± 1.3 to 2.4 ± 1.2, representing an improvement of 46.7% from baseline. The mean VAS for ankle pain reduced from 4.0 ± 1.8 to 1.8 ± 0.9, representing an improvement of 55.0%. Achieving this degree of pain reduction appears to be clinically relevant, since reductions in chronic pain intensity of at least 30% have been reported to reflect at least moderate clinically important changes [23]. The result of our study is very encouraging, as it continues to build on the existing data suggesting benefits from BoNT-A injections in the joints.

The exact mode of action of BoNT-A in OA has not been revealed completely. OA represents a complexity of pain conditions, including manifestations of both nociceptive and neuropathic mechanisms driven by joint pathophysiology and abnormal excitability in peripheral and central pain pathways [24-29]. The peripheral and central sensitizations may amplify the joint pain. It has been suggested that BoNT-A suppresses the secretion of neurotransmitters directly decreasing peripheral sensitization and indirectly decreasing central sensitization [30,31]. Recent studies also indicated a potential for inhibiting the release of mediators involved in nociception such as substance P, calcitonin gene related peptide and glutamate, which leads to a decrease in pain transmission and peripheral sensitization [32].

To our knowledge, this is also the first study that evaluate efficacy of a single hyaluronate injection plus rehabilitation exercise in patients with ankle OA. The mean AOS pain subscale reduced from 4.5 ± 1.1 to 2.5 ± 1.1 at 6-month follow-up, corresponding to an improvement of 44.4% from baseline. The mean VAS for ankle pain reduced from 3.9 ± 1.2 to 1.7 ± 1.1, representing an improvement of 56.4%. Achieving this degree of pain reduction suggests a success in chronic pain clinical trials, using the same criteria that pain reductions of at least 30% appear to reflect at least moderate clinically important changes [23]. We previously reported a mean AOS score reduction from 5.5 ± 2.1 prior to injection to 3.2 ± 1.9 points at six months after three weekly injections of hyaluronate in patients with Kellgren-Lawrence grade 2 or 3 ankle OA [13]. In another pilot study, we reported a mean AOS score reduction from 5.1 ± 1.9 to 2.4 ± 1.9 at the 6-month follow-up visit after five weekly injections of another hyaluronate formulation (ARTZ; Seikagaku Corporation, Japan) in patients with Kellgren-Lawrence grade 1 or 2 ankle OA [12]. Although pain and disability reduction was also documented in our current study, the results were difficult to compare with previous studies because of differences in the formulation, the number of injections, the radiographic severity, and the study design. In our current study, a single hyaluronate injection, offering similar clinical efficacy, allows a major compliance and convenience from the patients and reduces risks connected to intraarticular injection. This novel therapy seems promising and the optimal dose of hyaluronate and duration of rehabilitation exercise await further investigation. However, two treatments applied to the same group of patients, the improvement could have been due just to the rehabilitation component.

Balance is an important component of performance for transfer, ambulatory tasks and many daily activities. To date, there was only one case series that examine the effect of hyaluronate injections for ankle OA on balance [13]. Hubbard et al. reported significant impairments in mechanical and sensorimotor function in patients with ankle OA [33]. Pain associated with OA frequently leads to a reduced activity level and weakening of muscles, resulting in a secondary increase in instability. Reduced muscle strength and deficits in lower limb proprioception associated with OA could compromise effective and timely motor responses for maintaining balance [34,35]. In this study, we demonstrated that the two novel therapies are associated with improvement in pain and disability, as well as improvement in balance function. Although the mechanisms with balance improvement remain unknown, we believe that pain reduction might be one of the major contributing factors. Interestingly, the hyaluronate group improved more in SLS than the BoNT-A group at 1-month follow-up. We thought that the improvement could have been due to the rehabilitation component in the hyaluronate group.

This pilot study supports that a single injection of BoNT-A or one hyaluronate injection plus rehabilitation exercise may be valuable and attractive treatment option for ankle OA, especially when surgery is contraindicated or deferred due to age, comorbidities, or patient preference. The positive effects of the two therapeutic options in patients with ankle OA suggest that both are promising approaches worthy of serious clinical investigation.

Several limitations existed in this study. We recruited patients with Kellgren-Lawrence grade 2 ankle OA only, the results can not be generalized to other OA populations with different radiographic severity. The six-month trial period was relatively short, it is unclear how much longer the clinical benefits would have been maintained. We acknowledged that the hyaluronate group in our study received rehabilitation and the BoNT-A group did not, this may threaten the internal validity of the study. Due to economic and manpower concerns, we did not have sham control group (saline injection), sham injection plus rehabilitation group, or rehabilitation exercise only group. The improvements could be attributed to intraarticular injection, rehabilitation, non-intervention effects such as placebo or synergic effects of both treatments. It is difficult to address the relative contribution of each therapy procedure to the functional gains. This remained an interesting point important for further investigation. Further studies comparing BoNT-A injection with corticosteroid injections, different molecular weight hyaluronate, NSAIDs, or therapeutic exercise, as well as other potential combination therapy, may help determine the best overall treatment plan for patients with ankle OA. The cost-benefit ratio should be analyzed also.

## Conclusions

This study shows that both treatments with a single BoNT-A injection to the ankle joint or a single hyaluronate injection plus rehabilitation exercise were associated with improvements in pain, function and balance in patients with unilateral ankle OA. There were no significant differences in effectiveness between the 2 treatment groups. These effects are rapid at 2 weeks and can last for at least 6 months.

## Competing interests

The authors declare that they have no competing interests. The study was supported by a grant of VGHKS100-061 (an academic research fund from the hospital’s medical research council).

## Authors’ contributions

All authors contributed to the conception and design of this research. SS, YC, JC and JW performed the experiments. YC provided participants, JC and JW performed patient follow-up. HL and CH performed the statistical analysis. SS and CH interpreted data and drafted the manuscript. All authors read and approved the final manuscript.

## Supplementary Material

Additional file 1 Ankle rehabiliation exercise program. Click here for file
