# Supplementary material for: Efficacy of intraarticular botulinum toxin A and intraarticular hyaluronate plus rehabilitation exercise in patients with unilateral ankle osteoarthritis: a randomized controlled trial
Source: J Foot Ankle Res. 2014 Feb 6;7:9. doi: 10.1186/1757-1146-7-9 (PMC3922455; doi:10.1186/1757-1146-7-9)
Supplement: Additional file 1 — Ankle rehabiliation exercise program. [file 1757-1146-7-9-S1.doc]

**Ankle Rehabiliation Exercise Program**

(1) Heel cord stretching exercise and active ankle range of motion exercises for 10 minutes

(2) Proprioceptive exercises using balance board for 10 minutes

(3) Isometric exercises and strengthening exercises (using elastic band) for ankle muscles (including dorsiflexors, plantar flexors, invertors and evertors) for 10 minutes.

Hold each muscle contraction for 15 seconds and relax for 10 seconds.

Repeat each exercise for 6 times

Increase the number of repetitions as you get stronger.
